# Oxidants in Acute and Chronic Lung Disease

**DOI:** 10.4172/2165-7831.1000128

**Published:** 2014-09-11

**Authors:** Praveen Mannam, Anup Srivastava, Jaya Prakash Sugunaraj, Patty J Lee, Maor Sauler

**Affiliations:** 1 Pulmonary, Critical Care and Sleep Medicine, Yale University School of Medicine, New Haven, CT, USA; 2 Pulmonary and Critical Care Medicine, Geisinger Medical center, PA, USA

**Keywords:** Reactive oxygen species, Chronic obstructive pulmonary disease, Acute lung injury, Oxidative phosphorylation, Oxidant stress

## Abstract

Oxidants play an important role in homeostatic function, but excessive oxidant generation has an adverse effect on health. The manipulation of Reactive Oxygen Species (ROS) can have a beneficial effect on various lung pathologies. However indiscriminate uses of anti-oxidant strategies have not demonstrated any consistent benefit and may be harmful. Here we propose that nuanced strategies are needed to modulate the oxidant system to obtain a beneficial result in the lung diseases such as Acute Lung Injury (ALI) and Chronic Obstructive Pulmonary Disease (COPD). We identify novel areas of lung oxidant responses that may yield fruitful therapies in the future.

## Introduction

Oxygen is essential for complex biologic life. While unicellular organisms existed on this planet for nearly 4.5 billion years, a dramatic increase in the earth's oxygen 2.3 billion years ago permitted the emergence of multicellular organisms. This evolutionary step was fueled by the bio-energetic capabilities and signaling pathways that were enabled by this oxygen surge. A second increase in atmospheric oxygen 1.5 billion years later led to life forms of increasing complexity and the phylogeny of modern plant and animal species [1,2]. However, the co-opting of oxygen to create energy and sustain life is accompanied by potentially deleterious consequences. While oxygen is crucial for metabolism and certain enzymatic functions, it is also a source of highly reactive molecules that contribute to the pathologic consequences of oxidative stress in the lung. This article will discuss the role of oxidants generated by the mitochondria and the NADPH oxidase (Nox system) in the lung diseases of ALI and COPD. We will discuss the record of antioxidants in ameliorating these diseases and suggest future directions for research and possible therapeutic targets.

## ROS and Oxidative Stress

**Figure 1:**
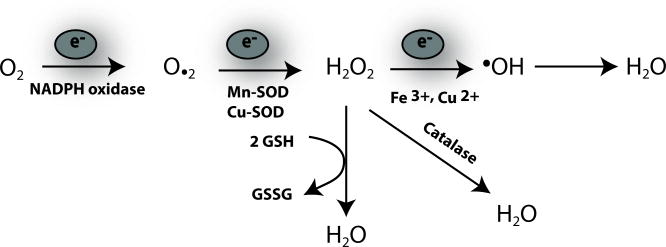
Biochemistry of ROS The first step in the formation of ROS is the gain of an electron by oxygen to form superoxide (O_2_·^−)^, a reaction that is catalyzed by NADPH oxidase. Further addition of electrons, mediated by manganese and copper superoxide dismutases (Mn-SOD, Cu-SOD), generates other forms of ROS such as hydrogen peroxide (H_2_O_2_). H_2_O_2_ is converted to hydroxyl radical (.OH) catalysed by Fe^3+^ and Cu^2+^. Antioxidant systems in the lung include the catalase system and the glutathione system both of which are complementary systems to reduce H_2_O_2_ to water. In the glutathione reaction reduced glutathione (GSH) is converted to glutathione disulfide (GSSG).

Reactive Oxygen Species (ROS) are a class of molecules that contain oxygen and readily react with bio-organic compounds. Many ROS possess one or more unpaired valence electrons (i.e. free radical), and all ROS can participate in reduction-oxidation (Redox) reactions (Figure 1).

These molecules are routinely generated from endogenous sources such as mitochondrial respiration and enzymatic reactions and the inherent instability of ROS allow for fast interactions with other molecules. Physiologic concentrations of ROS participate in major signaling pathways. As an example, the canonical transcription factors nuclear factor kappa-light-chain-enhancer of activated B cells (NF-κB), and activator protein 1 (AP-1) have redox sensitive cysteine residues that regulate their activity. When ROS occur in excess, acondition called oxidative stress, can lead to detrimental consequences.

These reactive molecules can form adducts with enzymes that result in protein misfolding and loss of function. ROS can also react with DNA with mutagenic consequences or oxidize lipids that can lead to the generation of carcinogenic compounds and the activation of inflammatory and apoptotic signaling pathways. Since ROS have the capacity to react in an indiscriminate manner with cellular components, an extensive range of antioxidant defenses have evolved to protect the cell from damage.

Antioxidant enzymes, non-enzymatic antioxidants, and transition metal binding proteins all interact to minimize collateral oxidation reactions that can lead to cellular dysfunction [3]. The distinction between oxidant injury and oxidant signaling is important as it is increasingly evident that non-specific quenching of all oxidants may have unintended consequences to important homeostatic cellular functioning. It is beyond the scope of this article to discuss the biology of ROS in detail and there are numerous exhaustive reviews on the topic of ROS (and related reactive nitrogen compounds) [4-6].

The lung is unique as it is constantly exposed to both exogenous and endogenous oxidating agents. Endogenous sources of free radical generation include mitochondrial leak, respiratory burst through NADPH oxidase, some enzymatic reactions like xanthine oxidation, and auto-oxidation reactions [3]. The lung is constantly exposed to exogenous sources include cigarette smoke, pollutants, UV-light, and ionizing radiations. Because virtually the entire blood circulation transits through its microvasculature with each cardiac cycle, oxidants generated elsewhere in the body can contribute to lung oxidative stress.

## The Mitochondria is Major Source of ROS

**Figure 2:**
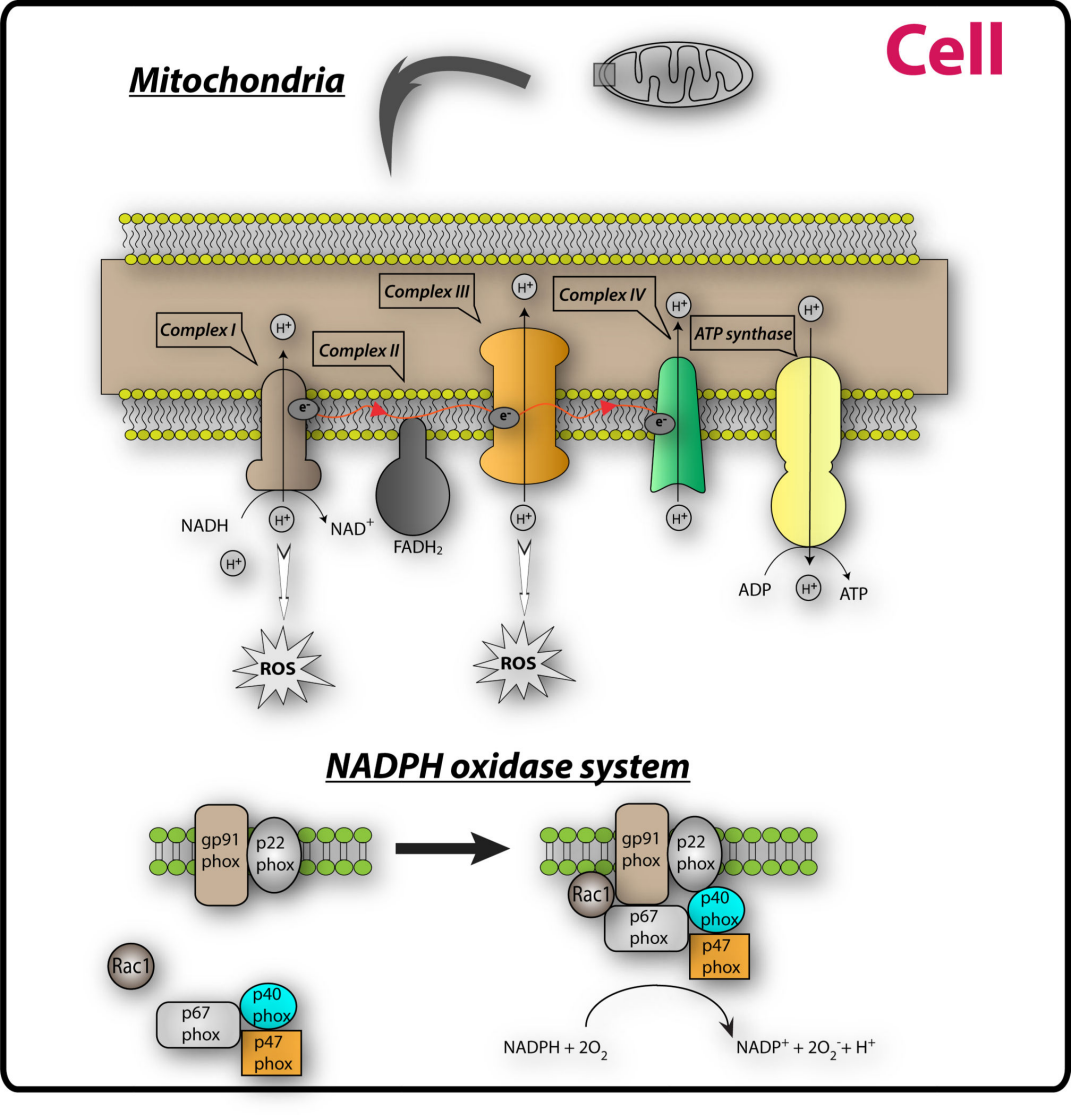
Major oxidant systems in the lung The mitochondria are a major source of ROS in the cell. ROS are generated as electrons constantly escape from the oxidative phosphorylation (OXPHOS) transport chain to generate superoxide. In the cytoplasm the NADPH oxidase (Nox) complex is another important source of ROS. The Nox complex is cluster of proteins that donate an electron from NADPH to molecular oxygen (O_2_) to produce superoxide (O_2_·^−^). The Nox system consists of 2 membrane bound subunits, gp^91^ phox and p^22^ phox. On stimulation the membrane bound units associate with a complex of Rac1, p^67^ phox, p^40^ phox and p^47^ phox, which then can transfer electrons from NADPH to oxygen to form the superoxide.

As the site of oxidative phosphorylation (OXPHOS), mitochondria are a major source of ROS in the cell. ROS are generated as electrons constantly escape from the transport chain to generate superoxide (O_2_·^−^) even under normal circumstances (Figure 2).

The steady-state concentration of O_2_·^−^ in the mitochondrial matrix is approximately five- to tenfold higher than that in the cytosol or nuclear space [7]. Most of the O_2_·^−^ generated by intact mammalian mitochondria in vitro is made by Complex I. This O_2_·^−^ produced is mainly on the matrix side of the inner mitochondrial membrane [8]. Additionally, Complex III is regarded as an important site of O_2_·^−^production, especially when mitochondrial respiration is repressed (Figure 2). O_2_·^−^ produced at this site appears at both sides of the inner mitochondrial membrane. Ubiquinone, a constituent of the mitochondrial respiratory chain, is considered as a key contributor in formation of O_2_·^−^ by Complex III [9]. The Q-cycle, oxidation-reduction of ubiquinone, is thought to be responsible for O_2_·^−^ formation. The superoxide dismutase causes dismutation of superoxide anions, which results in H_2_O_2_ production. Following interaction of H_2_O_2_ and O_2_·^−^ in a Haber-Weiss reaction, or Fe^2+^- (or Cu^2+^)-driven cleavage of H_2_O_2_ in a Fenton reaction, generates the highly reactive and toxic hydroxyl radical (.OH). Furthermore, monoamine oxidase (MAO), a flavin containing amine oxidoreductase localized on the outer mitochondrial membrane, is also an important mitochondrial source of ROS, in particular of H_2_O_2_ [10]. H_2_O_2_ crosses through mitochondrial membranes, thus MAO can contribute to an increase in the concentrations of ROS within the mitochondrial matrix and as well as cytosol. Mitochondria contribute 20%–30% of the cytosolic steady-state concentration of H_2_O_2_ [11]. There are other sources of the free radicals in mitochondria, such as mitochondrial p^66^(Shc), α-ketoglutarate dehydrogenase, besides redox cycling of redox-active molecules [12].

**Figure 3:**
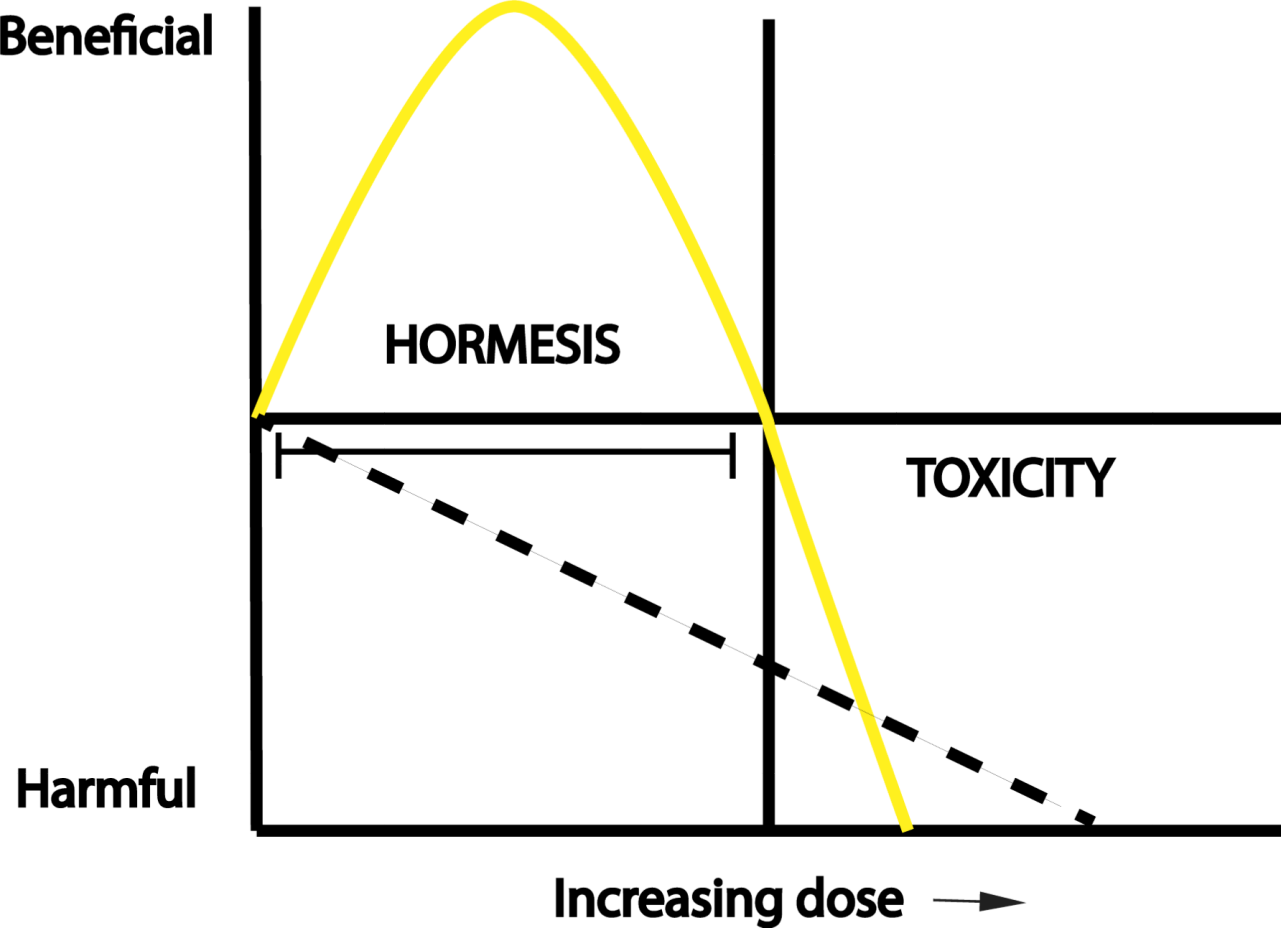
Concept of ROS hormesis Hormesis postulates that low doses of ROS are beneficial and have a physiologic role while increasing doses (yellow line) will cause toxicity. This is in contrast to the traditional view that all levels of ROS (dotted line) will have a harmful effect.

If the mitochondria are healthy and functional then ROS generation is low. However, as the components of the electron transport chain age or sustain damage from excessive ROS they will become dysfunctional leading to the vicious cycle of increased ROS production. To prevent this, mitochondria are constantly cleared by a process called mitophagy; while new mitochondria are simultaneously produced by mitochondrial biogenesis. Thus mitochondrial turnover is necessary to keep mitochondria young and healthy. While mitochondrial ROS has been frequently viewed as a deleterious by-product of the electron transport chain, there is an alternative view that a certain level of mitochondrial ROS is actually beneficial in promoting health, longevity, and stress resistance. This new concept of mitohormesis (Figure 3) postulates that this effect occurs as a result of transient mitochondrial ROS retrograde signaling to the nucleus [13]. There is no evidence to date that such a mechanism exists in the lung but as this may be an evolutionarily conserved mechanism of oxidant signaling and the lungs are constantly exposed to endogenous and exogenous oxidants, the implications for lungs are intriguing.

## The NADPH Oxidase System is a Cytoplasmic Source of ROS

In the cytoplasm the NADPH oxidase (Nox) complex is another important source of ROS. The Nox complex is a cluster of proteins that donate an electron from NADPH to molecular oxygen to produce superoxide. The Nox system consists of 2 membrane bound subunits, gp^91^ phox and p^22^ phox attached to either the plasma membrane or the endosome membrane in neutrophils and macrophages. On stimulation the membrane bound units associate with a complex of Rac1, p^67^ phox, p^40^ phox and p^47^ phox, which then can transfer electrons from NADPH to oxygen to form the superoxide (Figure 2).

There are different isoforms of Nox (termed Nox1-5 and Duox 1,2) depending on the variations of the major subunit (gp^91^phox) [14]. The prototypic Nox is Nox2, which is primarily expressed in neutrophils and macrophages and functions as a host defense mechanism against pathogens through the oxidative burst. Recent work has shown surprising versatility of the Nox family in other cell types such endothelial cells and also their function in non-infectious lung pathology [15,16].

The mitochondria, and Nox oxidant systems function cooperatively as initial mitochondrial ROS in necessary to prime the Nox system for sustained ROS production. Mitochondrial ROS achieves this by increasing the expression of the Nox isoforms and also by up regulating the Nox activity by activating the Rac1 component of the Nox complex [17,18]. This forms a richly textured network by which the lung is able to modulate and use oxidants for signaling. We need to understand this complexity better if we are to develop effective therapies against lung diseases, which are thought to result from excessive ROS generation, such as ALI and COPD. Of special interest among the Nox isoforms is Nox4, which localizes to the mitochondria and can increase ROS production in the mitochondria [19]. The interaction of Nox4 and mitochondrial ROS in lung is not yet clear but is a promising area of research.

## Lung Injury

Acute lung injury (ALI), or its severe form, acute respiratory distress syndrome (ARDS), are devastating clinical syndromes that are characterized by diffuse inflammation and consolidation. There is vascular permeability due to loss of the alveolar-capillary barrier and accumulation of protein-rich lung edema. The clinical manifestations include hypoxemia, bilateral infiltrates, and loss of lung compliance. The pathologic correlation of lung injury is diffuse alveolar damage, which involves various cell types such as inflammatory cells (neutrophils and macrophages), epithelial cells and endothelial cells. This process occurs as a result of dysregulated inflammation, activation of the coagulation cascade, altered permeability of alveolar endothelial and epithelial barriers, and oxidant stress. ALI and ARDS represent a broad clinical definition that encompasses complex types of injury from parenchymal and extra parenchymal etiology [20]. In addition, ALI and ARDS necessitate the use of supraphysiologic concentrations of oxygen, which in itself can exacerbate lung injury through hyperoxia mediated oxidant damage [21].

### Evidence for oxidant stress in lung injury

There are increased levels of oxidants in the broncho-alveolar lavage fluid (BAL) of patients with ARDS [22]. The source was thought to be primarily from the oxidative burst of alveolar macrophages and neutrophils but it is now recognized that the resident lung epithelial and endothelial cells are a significant source of ROS as well [23-25].

### The role of Mitochondria in lung injury

Sepsis and lung injury may be viewed as mitochondrial disorders. Mitochondria are essential hubs of innate immune signaling and are thought to be mediators of inflammatory responses [26–29] and the mitochondria are dysfunctional in patients with sepsis and lung injury [30,31]. The paradigm that organ failure in sepsis is due to failure of mitochondrial bioenergetics and ATP production is supported by recent studies [32–34]. Restoration of alveolar epithelial ATP through transfer of mitochondria from bone-marrow-derived stromal cells protected against LPS induced lung injury [35]. A study of the metabolome and proteome in septic patients found that mitochondrial functions such as the β oxidation and the citric acid cycle are major determinant of sepsis outcomes [36]. The plasma metabolome and lung transcriptome of monkeys infected with E. Coli infection showed that defects in β oxidation and mitochondrial metabolism lead to lung injury and death [37].

In addition dysfunctional mitochondria are sources of damage-associated molecular patterns (DAMPs) such as ROS, Mitochondrial transcription factor A (TFAM), which is a structural and functional homologue of high-mobility group protein B1 (HMGB1), ATP and mitochondrial DNA that initiate an inflammatory response further exacerbating organ injury[29,38–40]. Mitochondrial ROS in endothelial cells contribute to LPS induced lung injury through up regulation of the inflammatory transcription factors, NF-κB, AP-1 and Intercellular Adhesion Molecule 1 (ICAM-1) expression [41]. Our research indicates that MAP kinase kinase 3 (MKK3), a proximal activating kinase of the p38 MAPK is a novel regulator of mitochondrial function. MKK3-deficient mice are protected against sepsis and lung injury through the up regulation of mitochondrial biogenesis (formation of new mitochondria) and mitophagy (clearance of defective ROS producing mitochondria)[42]. Thus MKK3 may be an attractive therapeutic target in sepsis and lung injury. Another interesting role of mitochondrial function is evident from the field of inflammasomes. The inflammasome is a multiprotein complex that regulates the release of pro-inflammatory cytokines such as IL-1β in response to pathogens and endogenous danger signals. The inflammasome is associated with ARDS in humans and ventilator associated lung injury in mice [43,44]. We recently identified a protective role for Pink1 (a regulator of mitophagy) and NLRP3 (a component of the inflammasome complex) against hyperoxic lung injury [45]. Since mitochondrial function regulates inflammasome activation this pathway may be a target of therapies to reduce lung injury [28,46].

### The role of NADPH oxidase in lung injury

To date the isoforms Nox1, 2 and 4 have been implicated in the pathogenesis of ARDS. Hyperoxia increases Nox1 expression in both epithelial and endothelial cells of the lung and Nox1 deficiency is protective against epithelial and endothelial cell death [47]. Nox2 is the predominately expressed in macrophage and neutrophils. Nox derived from hematopoietic cells causes lung injury after ischemia-reperfusion [48] and Nox derived ROS from neutrophils are a major cause of lung injury and endothelial damage[49].

Nox4 is expressed in lung myofibroblasts and epithelial cells after bleomycin induced injury and inhibition of Nox4 suppressed the fibrogenic process after lung injury [50,51]. Nox4 has also been detected in lung endothelial cells where it generates ROS in response to hyperoxia [52]. Given the role of various Nox isoforms in the development of lung injury there is potential for therapeutic use. However barriers remain including the non-specific effect of the available Nox inhibitors against each of the isoforms and the concern that inhibition of Nox2 may impair the ability of phagocytic cells to clear infections [53].

### Therapeutic considerations in lung injury

There is hope that use of antioxidants will have a therapeutic impact in lung injury as the evidence suggests that oxidant stress plays a central role in the pathogenesis. However study results have been mixed, consistent with the poor performance of antioxidants in other diseases such as cancer and heart disease [54]. N-acetyl cysteine (NAC) is a small molecule thiol antioxidant that has been used extensively in the treatment of acetaminophen toxicity to regenerate glutathione.

NAC can reduce disulfide bonds and therefore neutralize oxidant species and is a widely used antioxidant. While animal studies using NAC have shown some effect in reducing lung injury, the use in human subjects was harmful with worse mortality when given 24hrs after admission in critically ill patients [55,56].

In another study of critically ill surgical trauma patients, antioxidant supplementation using alpha-tocopherol and ascorbic acid resulted in decreased incidence of multi organ failure and ICU length of stay [57]. Other anti-oxidants such as superoxide dismutase and catalase have shown therapeutic benefit in animal models of lung injury but have not been tested in humans [58]. In patients with acute lung injury randomized to supplementation with omega-3 fatty acids and antioxidants, investigators found that the patients receiving the supplementation did worse with higher mortality, fewer non-pulmonary organ failure-free and fewer vent free and ICU free days [59].

In the most comprehensive study to date administration of glutamine and antioxidants to critically ill patients with organ failure showed that no difference with the antioxidants but increased mortality in the group receiving glutamine [60].

Considering the fact that some of the signaling pathways set into motion by ROS may be beneficial, it is not surprising that blanket reduction of oxidants has given equivocal results so far. Furthermore, the optimal timing of anti-oxidant administration is not clear. Clearly in some situations ROS is necessary, as the oxidative burst of phagocytes is required to clear infection and transient, low levels of mitochondrial ROS are advantageous, as in the case of mitohormesis. Further research will be needed to delineate the timing and strategies to optimize antioxidant therapies.

**Figure 4:**
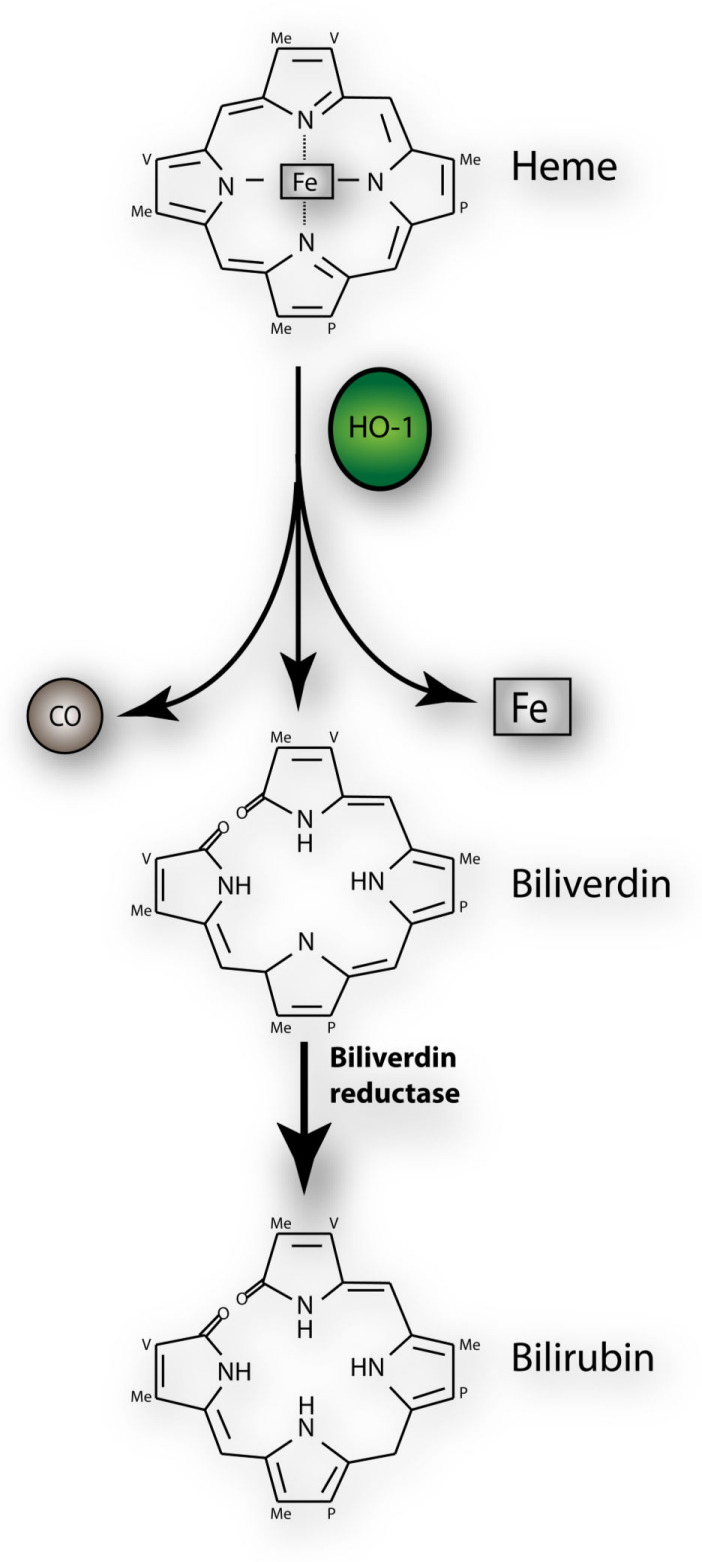
HO system Heme oxygenase (HO) is an enzyme that catalyzes the degradation of heme in the body to produce biliverdin, iron and the gas carbon monoxide (CO). Biliverdin is the subsequently converted to bilirubin by biliverdin reductase. HO^-1^ is a critical lung defense mechanism against oxidative stress and inflammation.

Recent research has highlighted the importance of the mitochondrial and Nox pathways in generation of ROS in the lung [16,61]. Use of specific Nox inhibitors and therapies against ROS-generating pathways may be of benefit in the treatment of lung injury. In the case of the mitochondria, strategies to improve mitochondrial turnover, through increased biogenesis and mitophagy, will be a fruitful area of future research. Another active area of research is Heme oxygenase -1 (HO-1). HO-1 is an enzyme that catalyzes the degradation of heme in the body to produce biliverdin, iron and the gas carbon monoxide (CO) (Figure 4) [62].

HO-1 is a critical lung defense mechanism against oxidative stress and inflammation. In animal studies, CO seems to mediate the antioxidant effect of HO-1 and clinical trials to increase the protective effects of HO-1 through CO inhalation are underway [63]. Intriguingly HO-1 has been described to be located in the mitochondria in the lung epithelial cells and protects against cigarette smoke induced damage [64]. This may mean that HO-1 has direct effect on mitochondrial function, which is an interesting avenue of research.

## COPD

Oxidative stress underlies the pathologic consequences of cigarette smoke. There are over 1016 oxidants per puff of cigarette smoke generated from over 4500 compounds [65,66]. Cigarette smoke recruits and activates neutrophils and macrophages, and these activated inflammatory cells generate significant quantities of endogenous oxidants through NADPH oxidases, mitochondrial oxidants, and various other peroxidase systems. The consequence of this chronic oxidant burden can induce pathologic hallmarks of COPD [67–70]. Airway inflammation, protease/anti-protease imbalance, premature lung aging, and cell death are all direct consequences of oxidative stress but are beyond the scope of this article and are reviewed elsewhere [71–74].

The use of exhaled breath condensates and other post-mortem studies have confirmed the presence of oxidative stress in the lungs from patients with COPD [75]. Notably, both direct measurements of oxidants, such as H_2_O_2_, or indirect measurements, of by-products of oxidative stress including 8-isoprostane and 4HNE, correlate with COPD severity and increase during acute exacerbations [76,77].

Simultaneously, an inability to mount an appropriate antioxidant defense is also associated with COPD [78].

The overwhelming evidence confirms the pathogenic role of oxidative stress in COPD. While the oxidative stress induced by cigarette smoke is well known, far less is known about the interaction between the exogenous oxidants of cigarette smoke and cellular sources of oxidant such as mitochondria and NADPH oxidases.

## The Role of Mitochondria in COPD

As compared to nuclear DNA, mitochondrial DNA is particularly sensitive to the effects of oxidative stress generated by cigarette smoke [79]. Mitochondrial DNA lies in close proximity to the electron transport system, which generates large quantities of ROS.

Cigarette smoke has been demonstrated to disrupt the electron transport chain leading to increased ROS production and mitochondrial dysfunction [80] Congruently, reduced levels of an inner mitochondrial protein prohibitin-1, postulated to serve as a chaperone for the electron transport chain, have been demonstrated in smokers and patients with COPD [81].

Studies of lung tissue from smokers have exhibited elevated measures of both mitochondrial DNA damage and mitochondrial DNA mutations when compared with nonsmokers. Evidence of mitochondrial dysfunction is not limited to the lungs. In platelets, smoking inhibits oxidative phosphorylation leading to increased mitochondrial ROS production [82].

Dysregulated mitochondrial ROS production has also been measured in both diaphragm and skeletal muscle of cigarette smokers and may explain the sarcopenia evident in patients with COPD [83,84]. The consequences of smoke induced mitochondrial dysfunction exacerbates two processes that contribute to COPD pathogensis [85,86]. Mitochondria are crucial regulators of programmed cell death and mitochondrial dysfunction may contribute to increased apoptosis in COPD [87].

Additionally, ROS generated as a consequence of mitochondrial dysfunction may compound tobacco smoke mediated oxidative stress [88,89]. Recent studies have demonstrated the therapeutic role of modifying mitochondrial viability in the treatment of COPD.

A recent publication suggests that the transfer of mitochondria from mesenchymal stem cells could attenuate cigarette smoke induced emphysema in rats [90]. Additionally, utilization of the mitochondrial division/mitophagy inhibitors can ameliorate cigarette smoke mediated cell death and murine emphysema [91]. This data supports the role of mitochondria and its contribution to disease pathogenesis.

## The Role of NADPH Oxidase in COPD

Increased airway inflammation is a hallmark of COPD and linked, in part, to airway recruitment of neutrophils [92]. Given that Noxs are activated in recruited neutrophils, one could surmise a role for Nox enzymes as one source of increased ROS in COPD. Interestingly, the differential role of Nox enzymes and their contribution to the development of COPD appears complex and poorly understood. While chronic cigarette smoke exposure can induce Nox1, mice lacking the Nox2 isoform, responsible for the oxidative burst in neutrophils, demonstrated increased susceptibility to cigarette smoke [93,94].

Differential regulation of Duox1 and Duox2 has also been demonstrated in the airway epithelium of individuals with COPD, healthy smokers and non-smokers [95]. However, this finding has yet to be replicated in the alveolar epithelium highlighting tissue-specificity in the pathogenic role of oxidative stress in COPD.

Moreover, Nox activation may be interwoven with innate immune signaling pathways. TLRs are the canonical pattern recognition receptor triggering the innate immune response. While increased airway inflammation is a hallmark of COPD, there is a paradoxical decrease in innate host defense in COPD [96].

Decreased TLR2 and TLR4 functioning has been demonstrated in patients with COPD which in part may underlie the increased susceptibility to airway colonization by pathogenic bacteria [97,98].

Concurrently, Nox isoforms have been demonstrated to regulate TLRs while simultaneously, certain TLR signaling cascades are Nox-dependent. An example of this complex interplay is the role of TLR4 and Nox3 in COPD. Nox3 is an isoform that is usually absent in normal, adult lungs, but can be induced in murine adult lung and lung endothelial cells, with the unexpected finding that Nox3 is regulated by TLR4. We found that TLR4 knockout mice develop spontaneous COPD due to increased synthesis of Nox 3, a phenotype that is partially reversed by concomitant knockdown of the Nox3 enzyme [15].

## Antioxidant Defense in COPD

Biologic responses to oxidative stress have been extensively studied. Glutathione, via its role as a reducing agent, is a critical mediator of host antioxidant defense in the lung airway. While total glutathione levels are not decreased in COPD, their levels may be inadequate given the increased presence of oxidants as a result of cigarette smoke [99]. Genetic polymorphisms in enzymes associated with glutathione synthesis, such as members of the glutathione S-transferase family, have also been implicated in the pathogenesis of COPD [100-103]. Similarly, genetic polymorphisms in the superoxide dismutase (SOD) family of antioxidant enzymes, Mn-SOD and Cu-SOD have also been associated with COPD [101,104,105].

Many antioxidant responses are controlled by Nuclear factor erythroid-2-related factor 2 (Nrf2). Nrf2 is an evolutionary conserved transcription factor that is sequestered and targeted for proteosomal degradation under basal conditions but results in the transcription of genes collectively associated with a cis acting binding site known as the antioxidant response element(ARE) [106,107]. During oxidative stress. Nrf2 is up regulated acutely during oxidative stress, but chronic cigarette smoke leads to a depletion of tissue Nrf2 [108]. Decreased Nrf2 mRNA and protein has been documented in tissue samples from patients with COPD and animal studies have confirmed that Nrf2 deficiency leads to cigarette smoke-induced emphysema. It has been suggested that this response is due to epigenetic changes as a consequence of histone deacetylase 2 (HDAC2) inhibition. Genome wide studies have confirmed SNPs in DJ1, a positive regulator of Nrf2, as associated with the development of disease [78]. There are multiple antioxidant and detoxifying genes that are modulated by the ARE. These include glutathione S-transferase, HO-1, and NADPH quinone oxidoreductase (Nqo1) [109]. Polymorphisms in other antioxidant genes encoding antioxidant enzymes such as HO-1 and epoxide hydrolase have all been associated with susceptibility to the development of COPD [101,110].

## Therapeutic Considerations in COPD

Despite the pathogenic role of oxidative stress in COPD, pharmacologic utilization of antioxidants in COPD has not demonstrated dramatic improvements in disease outcomes. Results from clinical trials with NAC have been mixed. A Cochrane meta-analysis has suggested a decrease in exacerbation with the use of NAC. While the large scale BRONCHUS trial demonstrated no benefit, a recent study of high dose NAC demonstrated a modest decrease in exacerbations and improvement in Forced Expiratory Flow 25% to 75% but no improvement in various subjective dyspnea scores [111,112]. Attempts at using glutathione directly via oral or nebulized therapy have been largely unsuccessful, as has therapy with the precursor of glutathione, glutamate [113]. Trials of SOD mimetics and NOX inhibiters have been discontinued, as has clinical development of myeloperoxidase inhibitors [114]. The Nrf2 activator CDDO-imidazolide and sulforaphane (found in broccoli) showed efficacy in the treatment of COPD in early trials and large scale trials are underway [115].

## Conclusion

The disappointing results from therapeutic drug trials in ALI and COPD should not dissuade us from recognizing the importance oxidative stress plays in the pathogenesis of pulmonary disease. Rather, it highlights an incomplete understanding of a crucial but complex biologic process. The classical paradigm of oxidative stress as a balance between oxidants and antioxidants is incomplete as this model fails to account for a multitude of subtleties in oxidant signaling. Oxidants and anti-oxidants are generated by a variety of systems and likely effect cellular functioning in diverse ways. Some of these systems contribute to homeostatic functioning, making blanket antioxidant therapy ineffective, at best, and possibly deleterious. Alternatively, varied cell types and biologic compartments have dissimilar regulation and responses to ROS. Further understanding of this complexity is necessary to determine how to deliver anti-oxidant therapy. Finally, while oxidant generation is critical to host defense, there is a limited understanding of how immunity regulates the oxidant/antioxidant balance and the subsequent pathologic consequence this imbalance. Our discoveries of TLR4-mediated Nox3 suppression and MKK3-induced mitochondrial oxidants provide some insight into previously unappreciated links between the immune system and oxidant generation but a myriad of questions remain. As a fundamentally protective response, signaling pathways downstream of innate immune activation may prime cells for the anticipatory increase in oxidative stress as a result of impending inflammation. Consequently, anti-apoptotic and anti-oxidant responses linked to innate immune functioning may be utilized to combat oxidant related diseases. The indiscriminant use of antioxidant therapy reinforces an incorrect belief that oxidants are uniformly deleterious. Instead we should focus on therapies to modulate oxidants in a highly targeted and controlled manner. While overwhelming evidence demonstrates that excessive oxidative stress contributes to the development of pulmonary disease, further understanding of this complex system will be required before effective therapy can be administered.
